# The Synergistic Inhibition of Coronavirus Replication and Induced Cytokine Production by Ciclesonide and the Tylophorine-Based Compound Dbq33b

**DOI:** 10.3390/pharmaceutics14071511

**Published:** 2022-07-21

**Authors:** Yue-Zhi Lee, Hsing-Yu Hsu, Cheng-Wei Yang, Yi-Ling Lin, Sui-Yuan Chang, Ruey-Bing Yang, Jian-Jong Liang, Tai-Ling Chao, Chun-Che Liao, Han-Chieh Kao, Jang-Yang Chang, Huey-Kang Sytwu, Chiung-Tong Chen, Shiow-Ju Lee

**Affiliations:** 1 Institute of Biotechnology and Pharmaceutical Research, National Health Research Institutes, Miaoli 35053, Taiwan; biology213@nhri.edu.tw (Y.-Z.L.); sandyshi720218@nhri.edu.tw (H.-Y.H.); janeway@nhri.org.tw (C.-W.Y.); jychang@nhri.edu.tw (J.-Y.C.); ctchen@nhri.edu.tw (C.-T.C.); 2 Institute of Biomedical Sciences, Academia Sinica, Taipei 115021, Taiwan; yll@ibms.sinica.edu.tw (Y.-L.L.); rbyang@ibms.sinica.edu.tw (R.-B.Y.); jjliang1234@yahoo.com.tw (J.-J.L.); jfliao@gmail.com (C.-C.L.); 3 Institute of Clinical Laboratory Sciences and Medical Biotechnology, College of Medicine, National Taiwan University, Taipei 10617, Taiwan; sychang@ntu.edu.tw (S.-Y.C.); iatoahc@gmail.com (T.-L.C.); r07424010@ntu.edu.tw (H.-C.K.); 4 National Institute of Infectious Diseases and Vaccinology, National Health Research Institutes, Miaoli 35053, Taiwan; sytwu@nhri.edu.tw

**Keywords:** COVID-19, ciclesonide, cytokine, IL-6, IL-8, MCP-1, MCP-3, OC43, SARS-CoV-2, tylophorine

## Abstract

Ciclesonide is an inhaled corticosteroid used to treat asthma and has been repurposed as a treatment for mildly ill COVID-19 patients, but its precise mechanism of action is unclear. Herein, we report that ciclesonide blocks the coronavirus-induced production of the cytokines IL-6, IL-8, and MCP-1 by increasing IκBα protein levels and significantly decreasing p65 nuclear translocation. Furthermore, we found that the combination of ciclesonide and dbq33b, a potent tylophorine-based coronavirus inhibitor that affects coronavirus-induced NF-κB activation a little, additively and synergistically decreased coronavirus-induced IL-6, IL-8, and MCP-1 cytokine levels, and synergistically inhibited the replication of both HCoV-OC43 and SARS-CoV-2. Collectively, the combination of ciclesonide and dbq33b merits consideration as a treatment for COVID-19 patients who may otherwise be overwhelmed by high viral loads and an NF-κB-mediated cytokine storm.

## 1. Introduction

The COVID-19 pandemic has been ongoing for more than two years, with 520.51 million people infected and over 6.29 million dead, as of 20 May 2022 (https://www.cdc.gov.tw/, accessed on 20 May 2022). The disease is caused by infection from severe acute respiratory syndrome coronavirus 2 (SARS-CoV-2), and its progression is usually complexed with a cytokine storm and/or organ dysfunction. Despite the deployment of numerous vaccines, SARS-CoV-2 continues to mutate and sweep the world [1] with waves of infections for which new therapeutic treatments are urgently needed.

Mild COVID-19 cases can be managed in the outpatient setting with an armamentarium of small molecule drugs, including the anti-virals Molnupiravir and Paxlovid, both of which were newly developed and authorized by the FDA, and the repurposed anti-depressant fluvoxamine [2]. Moderately ill COVID-19 patients, such as those with lower respiratory disease, can progress rapidly and should be closely monitored, and those with severe or critical illness at risk of rapid clinical deterioration need intensive monitoring in a hospital setting.

In addition to amplifying itself in an infected host, SARS-CoV-2 also induces cytokine production, often resulting in a cytokine storm whose mitigation is a potential COVID-19 treatment strategy, especially in critically ill patients [3]. Intervention strategies targeting the cytokine storm include traditional anti-inflammatories and immunosuppressives, e.g., corticosteroids such as dexamethasone and cyclosporine, as well as newly developed biologics, e.g., monoclonal antibodies targeting pro-inflammatory cytokines, and recombinant cytokines [4,5]. The current standard of care for hospitalized COVID-19 patients includes strategies to combat the cytokine storm such as dexamethasone and methylprednisolone [6,7,8,9,10], as well as those to address the replication of the virus itself [11,12], such as remdesivir. Any potential regimen that targets both the cytokine storm and viral replication merits further development.

In previous work, we established tylophorine-based dbq33b as a highly effective anti-viral and anti-inflammatory agent, targeting the viral N protein-associated ribonucleoprotein complex to inhibit replication of a broad spectrum of coronaviruses [13,14,15] and decrease the production of inflammatory TNFα, iNOS, and COX-II [16]. However, dbq33b has a small effect on NF-κB, which is activated by coronavirus infection [14] and plays a key role in cytokine storms [17,18]. We sought to combine dbq33b with an anti-NF-κB agent to optimize their therapeutic effects. One potential anti-NF-κB agent is ciclesonide, a corticosteroid that has been used to successfully treat mild COVID-19 patients in Japan [19]. However, although ciclesonide is known to exert anti-inflammatory effects, which presumably mitigate the COVID-19-associated cytokine storm, more studies are needed to elucidate the details of its mechanism of action. [19,20,21,22].

Herein, we report that the combination of ciclesonide and tylophorine-based dbq33b additively and synergistically blocks coronavirus-induced cytokine production by inhibiting NF-κB activation, and synergistically inhibits the replication of SARS-CoV-2 and HCoV-OC43 in infected cells.

## 2. Materials and Methods

### 2.1. Cells, Viruses, and Chemicals

HCT-8 colon adenocarcinoma cells (ATCC^®^ CCL-244™), MRC-5 lung fibroblasts cells (ATCC^®^ CCL-171™), and the OC43 strain of human coronavirus (HCoV-OC43, ATCC^®^ VR1558™) were purchased from American Type Culture Collection (ATCC), USA, and Vero E6 cells (BCRC number: 60476; derived from ATCC CRL-1586) from Bioresource Collection and Research Center (BCRC), Hsinchu, Taiwan. HCT-8 cells were grown at 37 °C under an atmosphere of 5% CO_2_ in Dulbecco’s modified Eagle medium (DMEM) (Cytiva, Marlborough, MA, USA, SH30022.02), supplemented with 1% penicillin/streptomycin (Biological Industries, Cromwell, CT, USA, 03-031-1B) and 10% fetal bovine serum (FBS) (Biological Industries, 04-001-1A). MRC-5 cells were cultured in Eagle’s Minimum Essential Medium (MEM) (Gibco, Grand Island, NY, USA, 11095-080) and 10% FBS (VWR, Radnor, PA, USA, 97068-085). HCoV-OC43 was propagated by inoculation of the HCT-8 cell line. Vero E6 cells were cultured in DMEM containing 10% FBS. SARS-CoV-2 TCDC#4 (hCoV-19/Taiwan/4/2020), a local isolate, was propagated on Vero E6 cells as described [13]. Ciclesonide (S4646, 99.46%, HPLC) was purchased from Selleckchem (Houston, TX, USA); dbq33b (2-ethyl-7, 10, 11-trimethoxy-1, 2, 3, 4-tetrahydrodibenzo [*f*, *h*]-isoquinolin-4-ol, >95%, HPLC) and biotinylated tylophorine (Bio-Ty, 95.87%, HPLC) were prepared as previously described [23]. For compound treatment studies, cells were cultured in a growth medium containing 2% FBS. The tested compounds were added to the wells 0.5 h prior to the addition of HCoV-OC43 at an MOI of 0.05. The resultant cells were harvested at 0.5, 1, 6, 24, 30 h.p.i. and subjected to western blot analysis, immunofluorescent assay, or RNA isolation for semi-quantitative RT-PCR analysis.

### 2.2. Biotinylated Tylophorine Pull-Down Assay

The cell lysates (600 μg) of HCoV-OC43-infected HCT-8 cells (MOI of 0.05) harvested at the indicated time points were incubated with 10 μM Biotin-X-SSE or biotinylated tylophorine at 4 °C for 4 h. Then, the associated complexes were pulled down by Dynabeads™ M-280 Streptavidin (Invitrogen, Dynal AS, Oslo, Norway, 11206D) at 4 °C for 1.5 h. The resultant pull-down complexes were subjected to RNA isolation for semi-quantitative RT-PCR analysis or western blot analysis.

### 2.3. Semi-Quantitative RT-PCR Analysis

Total RNAs were extracted using TRIzol™ Reagent (Invitrogen, 15596018) according to the manufacturer’s protocol, and reverse transcribed to cDNA using SuperScript™ IV reverse transcriptase (Invitrogen, 18090050) and oligo-dT primers. The primers used to amplify the PCR products were designed as follows: OC43-ORF1 (F: 5′-CCACAAGGAGCCTTTCATGT-3′, R: 5′- GCAACTGAACAACCTGAGCA-3′), OC43-ORFN (F: 5′-CCCAAGCAAACTGCTACCTCTCAG-3′, R: 5′-GTAGACTCCGTCAATATCGGTGCC-3′), IκBα (F: 5′- CCTGCAAAATCCTGACCTGG-3′, R: 5′-CTCATAACGTCAGACGCTGG-3′), IL-6 (F: 5′-GCATCTCAGCCCTGAGAAAG-3′, R: 5′-ACAGCTCTGGCTTGTTCCTC-3′), IL-8 (F: 5′-GATGCCAGTGAAACTTCAAGC-3′, R: 5′-TATTCTCTTGGCCCTTGGC-3′), MCP-1 (F: 5′- TCTCAAACTGAAGCTCGCACT-3′, R: 5′-TTTGGGACACTTGCTGCTG-3′), MCP-3 (F: 5′- AGCCTCTGCAGCACTTCTGT-3′, R: 5′-TCCTGGACCCACTTCTGTGT-3′), GAPDH (F: 5′- CCCTGGCCAAGGTCATCCAT-3′, R: 5′-CCAGTAGAGGCAGGGATGAT-3′). Gene expression levels are specified relative to that of the housekeeping gene GAPDH.

### 2.4. Western Blot Analysis

Western blotting analyses were performed as described [24]. Primary antibodies used for western blotting were anti-p-p65 (Ser536) (Cell Signaling, Danvers, MA, USA, 3033), anti-p65 (Cell Signaling, 8242), anti-p-IκBα (Ser32) (Cell Signaling, 2859), anti-IκBα (Cell Signaling, 9242), anti-GAPDH (Cell Signaling, 2118), anti-Caprin-1 (Proteintech, Rosemont, IL, USA, 15112-1-AP), anti-HCoV-OC43-nucleocapsid protein (anti-HCoV-OC43-N) (Merck Millipore, Temecula, CA, USA, Mab9013), and anti-vinculin (GeneTex, Irvine, CA, USA, GTX109749); their correspondent secondary antibodies used were horseradish peroxidase coupled anti-rabbit IgG antibody (PerkinElmer, Boston, MA, USA, NEF812001EA) or horseradish peroxidase coupled anti-mouse IgG antibody (GeneTex, GTX213111-01).

### 2.5. Immunofluorescent Assay

p65 nuclear translocation studies were carried out using 3 × 10^5^ MRC-5 cells seeded in 12-well plates. Twenty-four hours after seeding, cells were pretreated with compounds for 0.5 h prior to HCoV-OC43 infection at an MOI of 0.05. The resultant cells at 1 or 30 h.p.i. were then fixed with 4% formaldehyde, permeabilized with 100% methanol, blocked with 5% BSA, and stained with anti-p65 antibody (Cell Signaling, 8242), and CF^®^488A goat anti-rabbit IgG was used as secondary antibody (Biotium, Hayward, CA, USA, 20012) to afford fluorescent signal for later detection. Cell nuclei were stained with Hoechst dye (Invitrogen, H3569). Fluorescent signals were detected by the ImageXpress Micro XLS Wide field High-Content Screening System (Molecular Device) under “autofocus” function and the best z-offset. MetaXpress-MDCStore2.3 software (Molecular Device) was applied to count total cell numbers and quantitate the cell numbers that were defined with significant positive nuclear translocated p65 using the correlation coefficient of 0.5 as the threshold value. Accordingly, the percentages of cells with significant nuclear translocated p65 (NF-κB activation) were calculated. The module settings for cell classification used the Pearson’s correlation coefficient of pixel intensity of two stains in the entire cell region (nucleus + gap + cytoplasm), which is typically the robust method for classifying translocation as manufacturer’s instruction.

For the anti-coronaviral activity studies, an immunofluorescent assay was performed as described [5]. Anti-HCoV-OC43 N protein (Merck Millipore, Mab9013) and FITC-conjugated anti-mouse immunoglobulin (MP Biomedicals, Irvine, CA, USA, 55499) were used as the primary and second antibodies for anti-HCoV-OC43. Anti-SARS-CoV-2 N protein (Dr. An-Suei Yang of the Genomics Research Center, Academia Sinica, Taipei, Taiwan) and Alexa Fluor™ 488 goat anti-human IgG (Invitrogen, A-11013) were used as the primary and second antibody for anti- SARS-CoV-2.

### 2.6. Detection and Profiling of a Panel of 42 Human Cytokines Using a Cytokine Array

For 24 h, 2 × 10^6^ MRC-5 cells were seeded in a 10 cm dish and then infected with HCoV-OC43 at MOI 0.05 for 30 h. A mock infection control was also performed. Changes in the concentrations of 42 cytokines in the culture supernatants with or without HCoV-OC43 infection were evaluated using a commercial cytokine array (Abcam, Cambridge, UK, ab133997) as per the manufacturer’s recommendations.

### 2.7. ELISA for Individual Cytokine Measurements

Test supernatants were diluted as needed for the desired ELISA assays. The cytokine levels of IL-6, IL-8, MCP-1, MCP-3, ENA-78, and GRO-α in the culture supernatants of HCoV-OC43-infected MRC-5 cells at 30 h.p.i. were quantified using human cytokine enzyme-linked immunosorbent assay kits as per the manufacturer’s recommendations for IL-6 (arigo Biolaboratories, Hsinchu, Taiwan, ARG80110), IL-8 (arigo Biolaboratories, ARG80111), MCP-1 (arigo Biolaboratories, ARG80128), MCP-3 (arigo Biolaboratories, ARG82623), ENA-78 (Elabscience Biotechnology, Houston, TX, USA, E-EL-H0046), and GRO-α (Elabscience Biotechnology, E-EL-H0045).

### 2.8. Drug Combination Studies

Anti-coronaviral activities, e.g., viral replication and cytokine induction, were assessed using a drug dose–response matrix. The online tool SynergyFinder (https://synergyfinder.fimm.fi/, accessed on 23 March 2021) was used to calculate the average synergy scores. Synergy effects were calculated by ZIP synergy scores as follows: <−10 (antagonistic effect); −10 to 10 (additive effect); >10 (synergistic effect).

### 2.9. Statistical Analysis

The statistical significance between the two groups at the same time point or the same dose was evaluated by two-way ANOVA followed by Tukey’s multiple comparison test; ^#^, ^##^, and ^###^ are used to denote the statistical significance for *p* < 0.05, *p* < 0.01, and *p* < 0.001, respectively. Otherwise, the statistical significance was evaluated by the two-tailed unpaired Student’s *t*-test; *, **, and *** are used to denote the statistical significance for *p* < 0.05, *p* < 0.01, and *p* < 0.001, respectively.

## 3. Results

### 3.1. The Viral and Cellular Targets of Tylophorine-Based Compounds in HCoV-OC43-Infected Cells Were Identified and Confirmed

Tylophorine-based dbq33b directly targets a broad spectrum of coronaviruses to inhibit their replication [13,14,15]. However, it also targets cellular IκBα mRNA and caprin-1 protein-associated ribonucleoprotein complex in TGEV-infected ST cells, blocking IκBα protein translation/synthesis, and is therefore unable to inhibit virus-induced NF-κB activation [14], a major driver of the cytokine storm experienced by COVID-19 patients [3].

**Figure 1:**
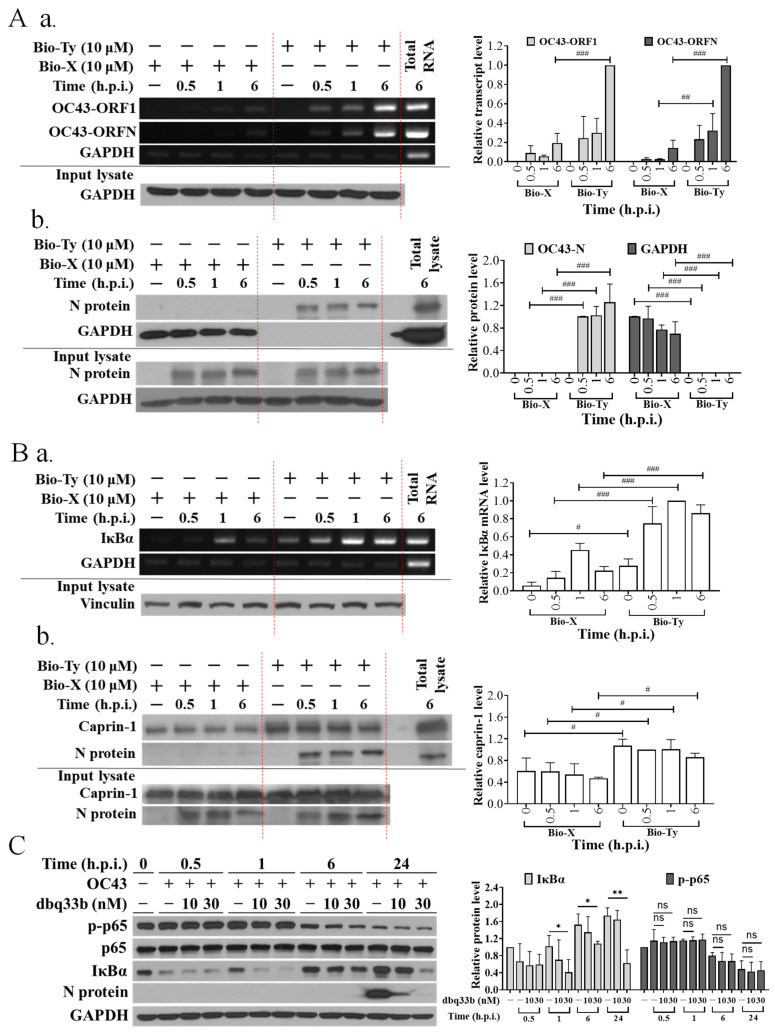
Viral and cellular targets of tylophorine-based compounds in HCoV-OC43-infected cells. (**A**) Tylophorine compounds interacted with HCoV-OC43 viral genomic RNA and nucleocapsid (N) protein. Biotinylated tylophorine interacted with HCoV-OC43 viral RNA (**a**) and N protein (**b**). (**B**) Tylophorine compounds interacted with host cellular IκBα mRNA and caprin-1 protein. Biotinylated tylophorine interacted with IκBα mRNA (**a**) and caprin-1 protein (**b**). (**C**) dbq33b diminished IκBα and viral antigen N protein levels in HCoV-OC43-infected HCT-8 cells. Lysates of HCoV-OC43-infected HCT-8 cells at the indicated post-infection time were subjected to pull-down experiments prior to RT-PCR or western analyses to examine the components (RNAs and proteins) in the association of biotinylated tylophorine with viral and cellular ribonucleoprotein complexes (**A**,**B**). The resultant lysates of HCoV-OC43-infected HCT-8 cells at the indicated post-infection time and treated dosage, respectively, were subjected to western analysis to examine the effect of tylophorine-based dbq33b on the viral and cellular protein levels of N protein, IκBα, p-p65, p65, and the internal control GAPDH (**C**). Shown results are representative of three independent experiments and AVE ± S.D. from three independent experiments. (ns: no significancy, * *p* < 0.05, ** *p* < 0.01 for student’s *t*-test; ^#^ *p* < 0.05, ^##^ *p* < 0.01, ^###^ *p* < 0.001 for two-way ANOVA test).

**Figure 2:**
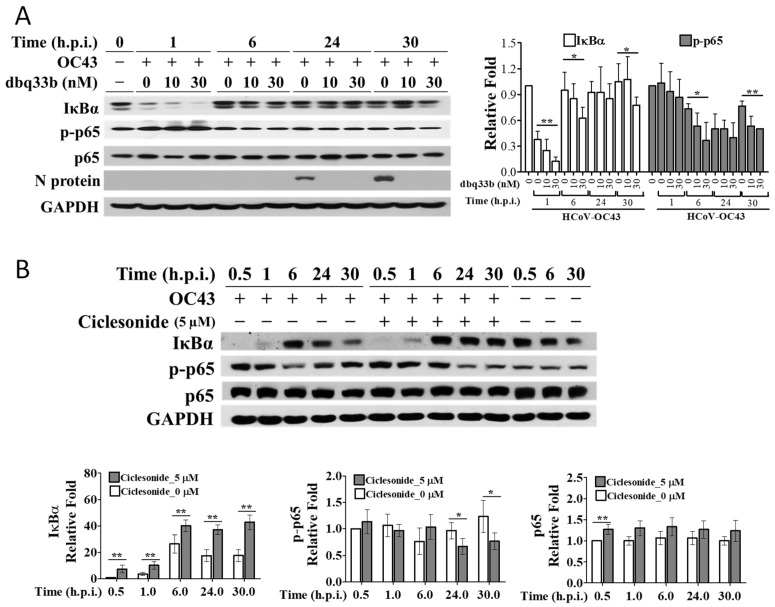

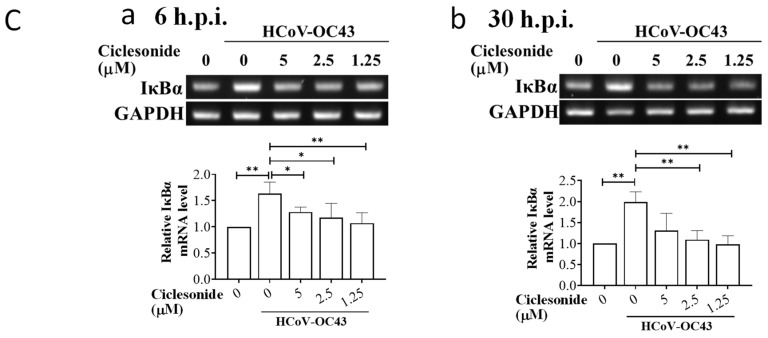
The effects of tylophorine-based dbq33b and ciclesonide on IκBα protein levels in HCoV-OC43-infected fetal lung fibroblast MRC-5 cells. (**A**) Effects of tylophorine-based dbq33b on the IκBα protein levels in HCoV-OC43-infected MRC-5 cells over a period of 30 h. (**B**) Ciclesonide treatment increased IκBα protein levels in HCoV-OC43-infected MRC-5 cells over a period of 30 h. (**C**) Ciclesonide decreased transcriptional levels of IκBα at 6 h.p.i. (**a**) and 30 h.p.i. (**b**). Uninfected and HCoV-OC43-infected (MOI: 0.05) MRC-5 cells treated with tylophorine-based dbq33b or ciclesonide (5 μM pretreatment for 1 h) were harvested at the indicated h.p.i. The resulting lysates were subjected to western analyses with the antibodies indicated (**A**,**B**). For transcriptional analyses, the uninfected and HCoV-OC43-infected (MOI: 0.05) MRC-5 cells treated with different concentrations of ciclesonide (0, 1.25, 2.5, 5 μM pretreatment for 1 h) were, respectively, harvested at 6 h.p.i. and 30 h.p.i. The TRIzol™ Reagent (Invitrogen, 15596018) was used to extract total RNA from the resultant cell lysates prior to performing RT-PCR using the specific primer pairs for the genes indicated. (**C**) Results shown are representative of three independent experiments and AVE ± S.D. from three independent experiments. (* *p* < 0.05, ** *p* < 0.01 for student’s *t*-test).

First, we confirmed that tylophorine-based compounds not only target the N protein-associated HCoV-OC43 viral genomic RNA complex (Figure 1A) to inhibit viral replication as indicated by N protein expression levels (Figure 1C and Figure 2A), but also the IκBα mRNA and caprin-1 protein-associated ribonucleoprotein complex (Figure 1B) to block IκBα protein translation/synthesis (Figure 1C and Figure 2A). The resultant diminished IκBα protein level is believed to preclude the inhibition of NF-κB activation by tylophorine-based dbq33b in human coronavirus OC43-infected HCT-8 cells (Figure 1C) since cytoplasmic IκBα protein degradation results in the release of its secured p-p65, which enters the nuclei causing NF-κB activation [25,26]. Accordingly, we searched for old drug(s) able to override the diminishing effect of tylophorine-based dbq33b on IκBα by inhibiting the coronavirus-induced NF-κB activation when administered in combination with dbq33b.

### 3.2. IκBα Protein Levels in HCoV-OC43-Infected MRC-5 Cells Were Increased by Ciclesonide and Decreased by Tylophorine-Based Dbq33b

Previously, we screened drugs covered by Taiwan Health Insurance for anti-viral activity in HCoV-OC43-infected HCT-8 cells to identify those potentially suitable for repurposing as an anti-SARS-CoV-2 agent to combat COVID-19 [5]. In this continued work, we found that ciclesonide, an inhaled corticosteroid used to treat hay fever, asthma, and allergic rhinitis, increased IκBα protein levels and decreased p-p65 protein levels (Figure 2B) over 30 h after the infection of MRC-5 cells with HCoV-OC43. This increase in IκBα protein levels caused by ciclesonide is in contrast to the decrease in levels seen in HCT-8 (Figure 1C) and MRC-5 cells (Figure 2A) infected with HCoV-OC43 after treatment with tylophorine-based dbq33b.

IκBα transcriptional levels were significantly increased at 6 h.p.i. and 30 h.p.i. in MRC-5 cells infected with HCoV-OC43. However, the treatment of infected MRC-5 cells with ciclesonide at the concentrations indicated caused IκBα transcriptional levels to decrease to amounts comparable with those in uninfected cells (Figure 2C). Hence, the increase in IκBα protein levels associated with ciclesonide treatment did not arise from transcriptional regulation. Therefore, we conclude that ciclesonide increased IκBα protein levels in HCoV-OC43-infected MRC-5 cells by slowing IκBα proteolysis, and not by increasing its transcription. The effect of this increase in IκBα was examined as follows.

### 3.3. Ciclesonide Decreased the p65 Nuclear Translocation/NF-κB Activation Induced by HCoV-OC43 Infection and Exerted a Synergistic Inhibition of NF-κB Activation in HCoV-OC43-Infected MRC-5 Cells When Combined with Tylophorine-Based Dbq33b

**Figure 3:**
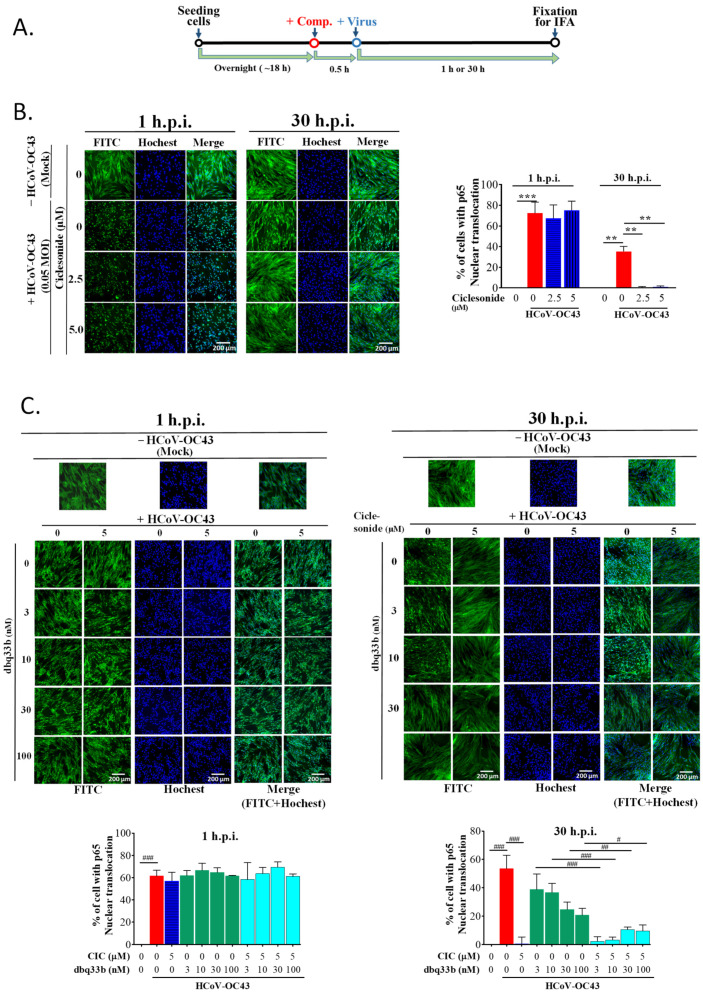
The effects of combined treatment with ciclesonide and tylophorine-based dbq33bon on the p65 nuclear translocation/NF-κB activation induced by HCoV-OC43 infection in MRC-5 cells. (**A**) Scheme for the sequence in which the HCoV-OC43 infection and compound treatments were performed. (**B**) The effect of ciclesonide on p65 translocation into nuclei induced by HCoV-OC43 infection in MRC-5 cells. (**C**) The effects of tylophorine-based dbq33b alone or in combination with ciclesonide on p65 translocation into nuclei induced by HCoV-OC43 infection in MRC-5 cells. After fixation, cells were treated with CF^®^488A goat anti-rabbit IgG for anti-p65 (green) and their nuclei visualized by staining with Hoechst (blue). Images were acquired using an ImageXpress Micro XLS Wide field High-Content Screening System under “autofocus” function and the best z-offset. Results shown are representative of three independent experiments and AVE ± S.D. from three independent experiments. (** *p* < 0.01, *** *p* < 0.001 for student’s *t*-test; ^#^ *p* < 0.05, ^##^ *p* < 0.01, ^###^ *p* < 0.001 for two-way ANOVA test). (Large images for (**B**,**C**) were provided in “Supplemental Data”).

The assay scheme for p65 nuclei translocation upon either HCoV-OC43 infection or compound treatment is shown in Figure 3A. First, the translocation of p65 into the nuclei of MRC-5 cells increased upon their infection with HCoV-OC43, reflecting the activation of NF-κB either at 1 h.p.i. or at 30 h.p.i. (Figure 3B). Next, we examined the effects of ciclesonide and tylophorine-based dbq33b, separately and in combination, on p65 nuclei translocation.

At 1 h.p.i., neither ciclesonide nor dbq33b nor their combination had any observable change effect on p65 translocation into the nuclei of HCoV-OC43-infected MRC-5 cells (Figure 3B,C). These results can be attributed to the significant reduction in IκBα protein levels caused by dbq33b at 1 h.p.i. (Figure 2A) and the relatively low levels of IκBα found after treatment by ciclesonide at 1 h.p.i. compared with those at 30 h.p.i. (Figure 2B).

On the other hand, as expected, ciclesonide significantly diminished p65 translocation into nuclei in HCoV-OC43-infected MRC-5 cells at 30 h.p.i. (Figure 3B), corresponding to an increase in IκBα protein levels along with infection time over a period of 30 h (Figure 2B). dbq33b moderately decreased p-p65 levels at 30 h.p.i. (Figure 2A), and p65 translocation into the nuclei of HCoV-OC43-infected MRC-5 cells decreased in a dose-dependent manner compared with that in untreated HCoV-OC43-infected MRC-5 cells at 30 h.p.i. (Figure 3C). This contrasts with the reported effect of dbq33b in TGEV-infected ST cells [14] and HCoV-OC43-infected HCT-8 cells, in which p-p65 levels were not decreased by tylophorine-based dbq33b (Figure 1C). Furthermore, when the combination of tylophorine-based dbq33b and ciclesonide was added to the HCoV-OC43-infected MRC-5 cells, the translocation of p65 into the nuclei was synergistically blocked at 30 h.p.i. (Figure 3C). Therefore, we conclude that ciclesonide increased IκBα protein levels (Figure 2B), suppressing NF-κB activation enough to override the diminishing effect of tylophorine-based dbq33b on IκBα (Figure 1C and Figure 2A). Thus, the combined treatment of ciclesonide and tylophorine-based dbq33b synergistically decreased NF-κB activation in HCoV-OC43-infected MRC-5 cells, as measured by the decrease in p65 nuclear translocation at 30 h.p.i. (Figure 3C).

### 3.4. HCoV-OC43 Infection Increased Production of IL-8, IL-6, and MCP-1 in MRC-5 Cells

**Figure 4:**
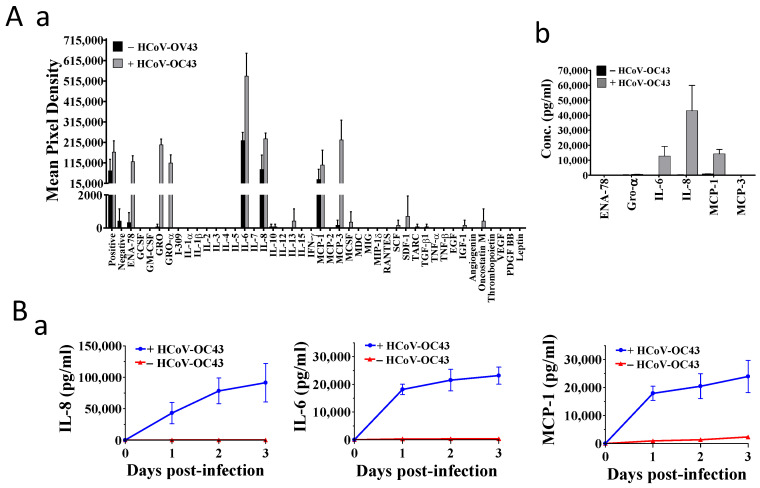

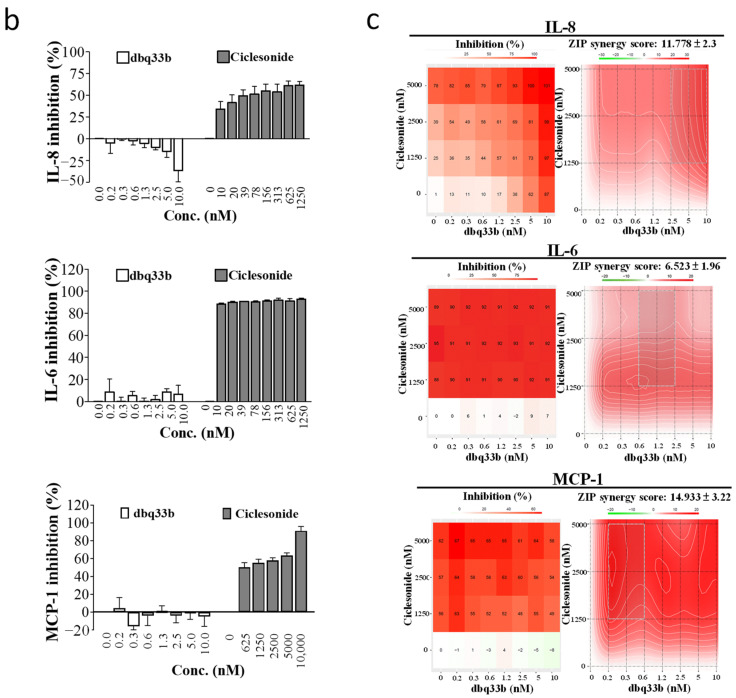
The effects of ciclesonide and tylophorine-based dbq33b, respectively, alone or in combination on the cytokine productions in HCoV-OC43-infected MRC-5 cells. (**A**) IL-8, IL-6, and MCP-1 were highly induced and produced in MRC-5 cells upon HCoV-OC43 infection at 30 h.p.i. (**a**) The changes in culture supernatant levels of 42 cytokines with or without HCoV-OC43 infection were evaluated using a cytokine array (Abcam, ab133997) from HCoV-OC43-infected cells and control cells at 30 h.p.i. (**b**) ELISA kits with high specificity as indicated were used to verify and evaluate the identified cytokine productions from (**a**). (**B**) The effects of ciclesonide and tylophorine-based dbq33b, separately or in combination, on the cytokine productions of IL-6, IL-8, and MCP-1. (**a**) IL-8, IL-6, and MCP-1 were highly produced in MRC-5 cells upon HCoV-OC43 infection over a period of three days. (**b**) The respective dosage effects of ciclesonide and tylophorine-based dbq33b on the production of IL-8, IL-6, and MCP-1 in HCoV-OC43-infected MRC-5 cells. Results shown are representative of three independent experiments and AVE ± S.D. from three independent experiments. (**c**). The effect of the combination of ciclesonide and tylophorine-based dbq33b on the production of IL-6, IL-8, and MCP-1 in HCoV-OC43-infected MRC-5 cells at 30 h.p.i. Shown synergy scores are AVE ± S.D. from three independent dose-matrix experiments analyzed via the online SynergyFinder (https://synergyfinder.fimm.fi/, accessed on 30 March 2021).

Next, we examined the effect of HCoV-OC43 infection on NF-κB activation and cytokine production in MRC-5 cells, which are capable of producing cytokines upon infection of HCoV-OC43 [5]. A 42-target human cytokine protein array (Abcam, ab133997), which included most of the cytokines reported in severely ill COVID-19 patients, was used to survey the cytokines induced by HCoV-OC43 in MRC-5 cells. As shown in Figure 4(Aa)), significant amounts of IL-6, IL-8. ENA-78, GRO, GRO-α, MCP-1, and MCP-3 were detected in the supernatants of HCoV-OC43-infected MRC-5 cells at 30 h.p.i. IL-6, IL-8, and MCP-1 were also detected in the supernatants of uninfected MRC-5 cells but in much lower amounts.

A series of ELISA kits with high specificity to individually detect these cytokines were used to validate these findings (Figure 4(Ab)). Neither ENA-78, GRO, GRO-α, nor MCP-3 were detected in significant amounts in either infected or uninfected cells, whereas the production of IL-6, IL-8, and MCP-1 was increased by HCoV-OC43 infection (Figure 4(Ab)). These findings are consistent with a study that profiled and ranked the levels of cytokines of COVID-19 patients with high mortality and identified IL-8, IL-6, and MCP-1 as the second, third, and fourth most important cytokines for distinguishing between ICU and non-ICU patients; MCP-3 was ranked first [27]. Hence, the gene expressions of IL-8, IL-6, MCP-1, and MCP-3 induced by HCoV-OC43 in MRC-5 cells were also examined and illustrated as follows.

### 3.5. Ciclesonide Significantly Inhibited HCoV-OC43-Induced Cytokine Production in Infected MRC-5 Cells and Exerted an Additive and Synergistic Inhibition in Combination with Tylophorine-Based Dbq33b

Since ciclesonide suppressed NF-κB activation by HCoV-OC43 in MRC-5 cells (Figure 3), we also examined the effects of ciclesonide on the protein and transcription levels of IL-6, IL-8, and MCP-1 by ELISA and RT-PCR, respectively. The top four highest scoring cytokines (MCP-3, IL-8 IL-6, and MCP-1) reported in COVID-19 patients with high mortality [27] are all transcriptionally regulated by NF-κB [17,18,28]. Therefore, the effects of viral infection and compound treatment on the transcriptional levels of MCP-3 were also examined.

At 30 h.p.i. with HCoV-OC43, the expressions of IL-6, IL-8, and MCP-1 were all up-regulated in MRC-5 cells. This up-regulation was profoundly suppressed by ciclesonide (Figure 4(Bb)) but not tylophorine-based dbq33b (Figure 4(Bb)). However, when the effects of ciclesonide and tylophorine-based dbq33b on the abovementioned cytokines were examined in matrix dose–response experiments, we found they synergistically suppressed IL-8 and MCP-1 production and additively suppressed IL-6 production at 30 h.p.i. (Figure 4(Bc) and Table 1).

**Figure 5:**
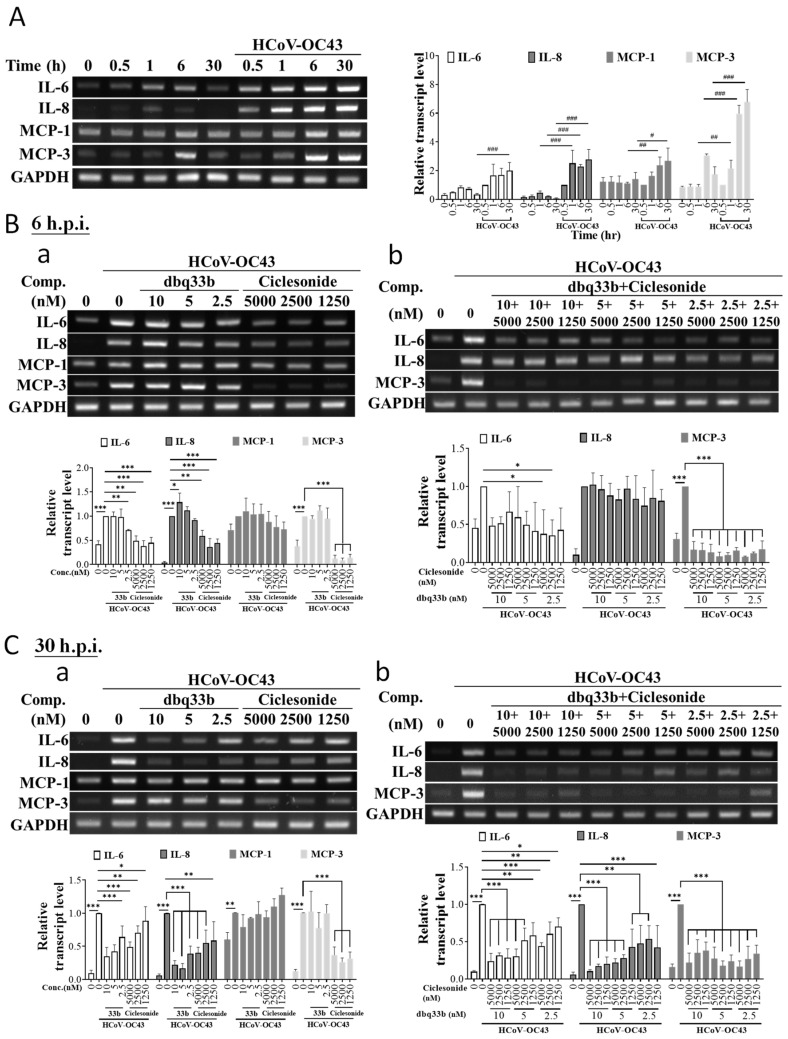
The effects of ciclesonide and tylophorine-based dbq33b alone or in combination on the transcriptional levels of cytokines induced by HCoV-OC43 in MRC-5 cells. (**A**) Transcriptions of cytokines, MCP-3, IL-8, IL-6, and MCP-1, were highly up-regulated in HCoV-OC43 in MRC-5 cells. (**B**) The effects of ciclesonide and tylophorine-based dbq33b, respectively (**Ba**), or in combination (**Bb**) on the up-regulated transcriptions of cytokines IL-6, IL-8, MCP-1, and MCP-3 at 6 h.p.i. (**C**). The effects of ciclesonide and tylophorine-based dbq33b respectively (**Ca**), or in combination (**Cb**) on the up-regulated transcriptions of cytokines IL-6, IL-8, MCP-1, and, MCP-3 at 30 h.p.i. The cells, in HCoV-OC43-infected MRC-5 (0.05 MOI) at 6 or 30 h.p.i. treated with vehicle (0.5% DMSO) or compounds as indicated, were subjected to TRIzol reagent for total RNA isolation and then RT-PCR for analysis of gene transcriptional expression of MCP-3, IL-8, IL-6, and MCP-1. Results shown are representative of three independent experiments and AVE ± S.D. from three independent experiments. (* *p* < 0.05, ** *p* < 0.01, *** *p* < 0.001 for student’s *t*-test; ^#^ *p* < 0.05, ^##^ *p* < 0.01, ^###^ *p* < 0.001 for two-way ANOVA test).

Whereas the gene expressions of IL-6, IL-8, and MCP-1 were all profoundly induced upon the infection of HCoV-OC43 over a period of 30 h after infection (Figure 5A), only the gene expressions of IL-6 and IL-8, but not MCP-1, were suppressed by ciclesonide treatment in a dose-dependent manner as examined either at 6 h.p.i. or 30 h.p.i. (Figure 5(Ba,Ca)). In contrast, tylophorine-based dbq33b significantly suppressed the gene induction of IL-6 and IL-8 at 30 h.p.i. (Figure 5(Ca)) but not 6 h.p.i. (Figure 5(Ba)) and insignificantly inhibited MCP-1 gene expression at both 6 h.p.i. (Figure 5(Ba)) and 30 h.p.i. (Figure 5(Ba)). Finally, when the combined effects of ciclesonide and tylophorine-based dbq33b on the gene expression of the abovementioned cytokines were further examined, it was found that they significantly suppressed IL-6 and IL-8 expressions at 30 h.p.i. (Figure 5(Cb)), but at 6 h.p.i had a reduced effect on IL-6 and little effect on IL-8 (Figure 5(Bb)).

Although ciclesonide was unable to inhibit the up-regulation of the MCP-1 gene upon HCoV-OC43 infection (Figure 5(Ba,Ca)), it nevertheless profoundly diminished MCP-1 protein levels (Figure 4B). Moreover, in addition to MCP-1, ciclesonide was also found to inhibit the gene expression of MCP-3, another target gene of NF-κB [28], both when used alone and when combined with tylophorine-based dbq33b (Figure 5B,C). However, treatment with dbq33b alone did not significantly affect MCP-3 gene expression levels in infected cells at either 6 h.p.i. or 30 h.p.i. (Figure 5B,C)

Therefore, we conclude that ciclesonide can down-regulate NF-κB regulated genes and their corresponding encoded proteins, and synergistically exert these inhibitory effects in concert with tylophorine-based dbq33b.

### 3.6. Tylophorine-Based Dbq33b and Ciclesonide Synergistically Inhibited HCoV-OC43 Replication in MRC-5 Cells and SARS-CoV-2 Replication in Vero E6 Cells

**Figure 6:**
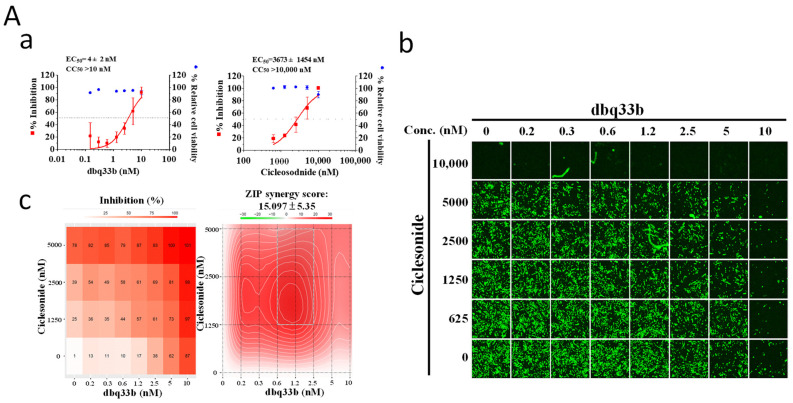

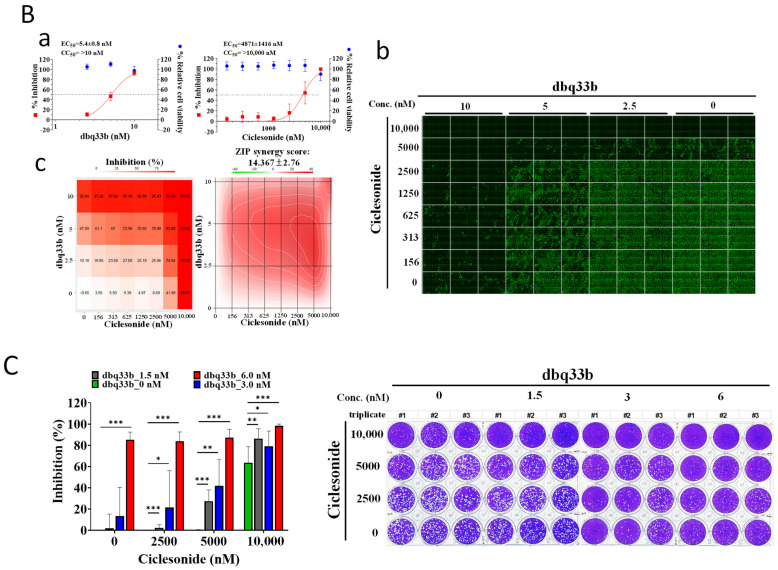
Combined treatments of ciclesonide and tylophorine-based dbq33b synergistically inhibited coronaviral replication. (**A**) Combined treatment of ciclesonide and tylophorine-based dbq33b synergistically inhibited HCoV-OC43 infection/replication in human fetal lung fibroblast MRC-5 cells assayed by IFA. (**B**) Combined treatment of ciclesonide and tylophorine-based dbq33b synergistically inhibited SARS-CoV-2 infection/replication in Vero E6 cells assayed by IFA. (**C**) Combined treatment of ciclesonide and tylophorine-based dbq33b synergistically reduced infectious SARS-CoV-2 viral loads as determined by plaque formation assays. IFAs were performed with antibody against viral N protein (green) in HCoV-OC43-infected MRC-5 (0.05 MOI) at 30 h.p.i. treated with vehicle (0.5% DMSO) or compounds as indicated. Nuclei (blue) were counterstained with Hoechst dye and used to determine the relative cell viability by using the number of nuclei in vehicle control as 100%. The Methods used for IFA and plaque assays for SARS-CoV-2 infectivity in Vero E6 cells were performed and analyzed as described [5]. The effects of single treatments within indicated concentrations were shown as AVE ± S.D. from three independent experiments (**Aa**,**Ba**,**C**). IFA or plaque formation images (**Ab**,**Bb**,**C**) shown are representative of three independent experiments. (* *p* < 0.05, ** *p* < 0.01, *** *p* < 0.001). Shown synergy scores are AVE ± S.D. from three independent experiments (**Ac**,**Bc**) analyzed via the online SynergyFinder (https://synergyfinder.fimm.fi/, accessed on 23 March 2021).

Tylophorine-based dbq33b is a highly potent inhibitor of coronavirus replication with an EC_50_ of 4 ± 2 nM against HCoV-OC43 (Figure 6A) but exerts an insignificant inhibitory effect on the expressions of IL-8, IL-6, or MCP-1 (Figure 4 and Figure 5). On the contrary, ciclesonide was a minimally potent inhibitor of HCoV-OC43 with an EC_50_ of 3673 ± 1454 nM (Figure 6A) in MRC-5 cells but exhibited a significant inhibitory effect on the production of IL-8, IL-6, and MCP-1 (Figure 4). Nonetheless, the combination of tylophorine-based dbq33b and ciclesonide exerts a synergistic effect against HCoV-OC43 replication in infected MRC-5 cells with a synergy score of 15.1 ± 5.4 (Figure 6A and Table 2) and SARS-CoV-2 in Vero E6 cells with a synergy score of ~14.4 ± 2.8 (Figure 6B and Table 2) as assayed by IFA, respectively, with a matrix dose response combination. Plaque formation assays also showed the combination to significantly diminish viral loads of SARS-CoV-2 in Vero E6 cells (Figure 6C).

## 4. Discussions and Conclusions

The cytokine storm experienced by SARS-CoV-2-infected patients is characterized by high levels of inflammatory cytokines and immune cell hyperactivation, and is strongly associated with COVID-19 disease severity and mortality [27,29,30]. The excess production of inflammatory factors including inflammatory cytokines [29,31,32] is a direct result of coronavirus-induced NF-κB activation.

Using a 48-plex cytokine screen, the top four cytokines most highly expressed by 181 COVID-19 patients were found to be MCP-3, IL-8, IL-6, and MCP-1, of which two, MCP-3 and IL-8, have been correlated with ICU admission and mortality [27]. MCP-3 and IL-6, along with some other cytokines, have been identified as potential predictors for COVID-19 disease progression, mortality, or severity [33,34,35].

Ciclesonide is a corticosteroid used to suppress inflammation. We found that ciclesonide effectively elevated IκBα protein levels, blocking p65 translocation into nuclei, and inhibiting NF-κB activation. This significantly reduced the protein expression levels of MCP-1, IL-6, and IL-8 and the gene expression levels of MCP-3, IL-6, and IL-8 in HCoV-OC43-infected cells (Figure 4 and Figure 5), findings which may establish ciclesonide as an effective treatment for mild cases of COVID-19 [19].

In addition to potently blocking coronaviral replication [13,14,15], tylophorine-based dbq33b also off-targets the ribonucleoprotein complex of IκBα mRNA and caprin-1 protein to block IκBα protein translation and synthesis, and is therefore incapable of inhibiting NF-κB activation in TGEV-infected ST cells [14]. Hence, drug(s) capable of blocking NF-κB activation by elevating IκBα protein levels could be well suited to combating the NF-κB-induced cytokine storm in COVID-19 patients when given in combination with dbq33b.

Lastly, the combined treatment of ciclesonide and dbq33b effectively inhibited NF-κB activation (Figure 3C), additively and synergistically diminishing NF-κB-regulated cytokine production (e.g., IL-8, IL-6, and MCP-1) or gene expression (e.g., MCP-3, IL-8, and IL-6) (Figure 4 and Figure 5), all of which are associated with high mortality in hospitalized COVID-19 patients [27,35]. They also synergistically blocked viral replication (Figure 6). Previous studies showed that tylophorine compounds inhibit coronaviral replication not only in a preventive manner (as in pretreatment) but also work therapeutically (as in post-treatment) [15]. In addition, ciclesonide has been therapeutically used in mild COVID patients [19]. Therefore, we conclude that the combined treatment of ciclesonide and dbq33b is a potential treatment regimen for COVID-19, simultaneously targeting both the host factors and SARS-CoV-2 itself. Drugs with a similar mechanism of action to ciclesonide are also candidates for development as a treatment regimen for COVID-19, in combination with dbp33b.

In conclusion, ciclesonide and dbq33b synergistically target coronavirus and NF-κB, respectively, effectively suppressing both viral replication and cytokine expression to potentiate anti-viral activity and mitigate a cytokine storm. Such a regimen therefore merits further development as a therapeutic to treat COVID-19 patients.

## Figures and Tables

**Table 1:** The additive and synergistic inhibition of IL-8, IL-6, and MCP-1 induction in HCoV-OC43-infected MRC-5 cells after treatment with the combination of ciclesonide and dbq33b.

Cytokine Inhibition	Synergy Score	Most Synergistic Area Score	Method
IL-8	11.78 ± 2.30 *	16.28	ZIP
IL-6	6.52 ± 1.96	6.84	ZIP
MCP-1	14.93 ± 3.22	15.42	ZIP

* AVE ± S.D. of synergy scores from three independent experiments. Synergy scores were calculated and analyzed by SynergyFinder, ZIP method.

**Table 2:** The synergistic inhibition of coronaviral replication in HCoV-OC43-infected MRC-5 cells and SARS-CoV-2-infected Vero E6 cells after treatment with the combination of ciclesonide and dbq33b.

CoV Inhibition/Cell Line	Synergy Score	Most Synergistic Area Score	Method
HCoV-OC43/HCT-8	15.10 ± 5.40 *	16.28	ZIP
SARS-CoV-2/Vero E6	14.37 ± 2.76	18.38	ZIP

* AVE ± S.D. of synergy scores from three independent experiments. Synergy scores were calculated and analyzed by SynergyFinder, ZIP method.

## Supplementary Materials

The following supporting information can be downloaded at: https://www.mdpi.com/1999-4923/14/7/1511/s1. Large images for Figure 3B,C were provided in “supplemental data”.
