# Correction: Lee et al. The Synergistic Inhibition of Coronavirus Replication and Induced Cytokine Production by Ciclesonide and the Tylophorine-Based Compound Dbq33b. *Pharmaceutics* 2022, *14*, 1511

**DOI:** 10.3390/pharmaceutics15020342

**Published:** 2023-01-19

**Authors:** Yue-Zhi Lee, Hsing-Yu Hsu, Cheng-Wei Yang, Yi-Ling Lin, Sui-Yuan Chang, Ruey-Bing Yang, Jian-Jong Liang, Tai-Ling Chao, Chun-Che Liao, Han-Chieh Kao, Jang-Yang Chang, Huey-Kang Sytwu, Chiung-Tong Chen, Shiow-Ju Lee

**Affiliations:** 1 Institute of Biotechnology and Pharmaceutical Research, National Health Research Institutes, Miaoli 35053, Taiwan; 2 Institute of Biomedical Sciences, Academia Sinica, Taipei 115021, Taiwan; 3 Institute of Clinical Laboratory Sciences and Medical Biotechnology, College of Medicine, National Taiwan University, Taipei 10617, Taiwan; 4 National Institute of Infectious Diseases and Vaccinology, National Health Research Institutes, Miaoli 35053, Taiwan

**Changes to “authors who contributed equally”**

In the original publication [1], the authors who had “contributed equally” were not listed. The corrected statement appears here. The authors state that the scientific conclusions are unaffected. This correction was approved by the Academic Editor. The original publication has also been updated.
