# Supplementary figures and images for: The Synergistic Inhibition of Coronavirus Replication and Induced Cytokine Production by Ciclesonide and the Tylophorine-Based Compound Dbq33b
Source: Pharmaceutics. 2022 Jul 21;14(7):1511. doi: 10.3390/pharmaceutics14071511 (PMC9325102; doi:10.3390/pharmaceutics14071511)

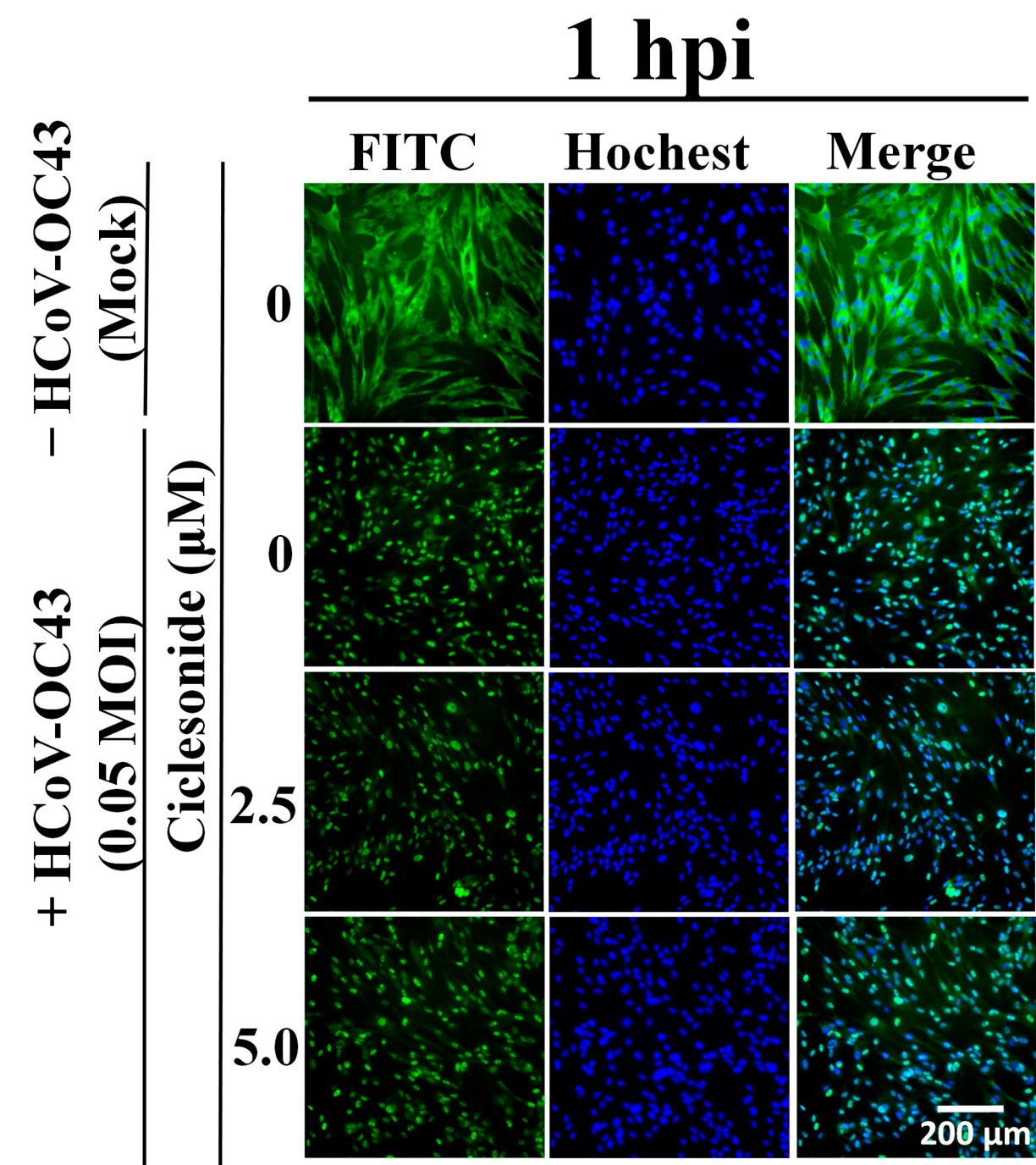

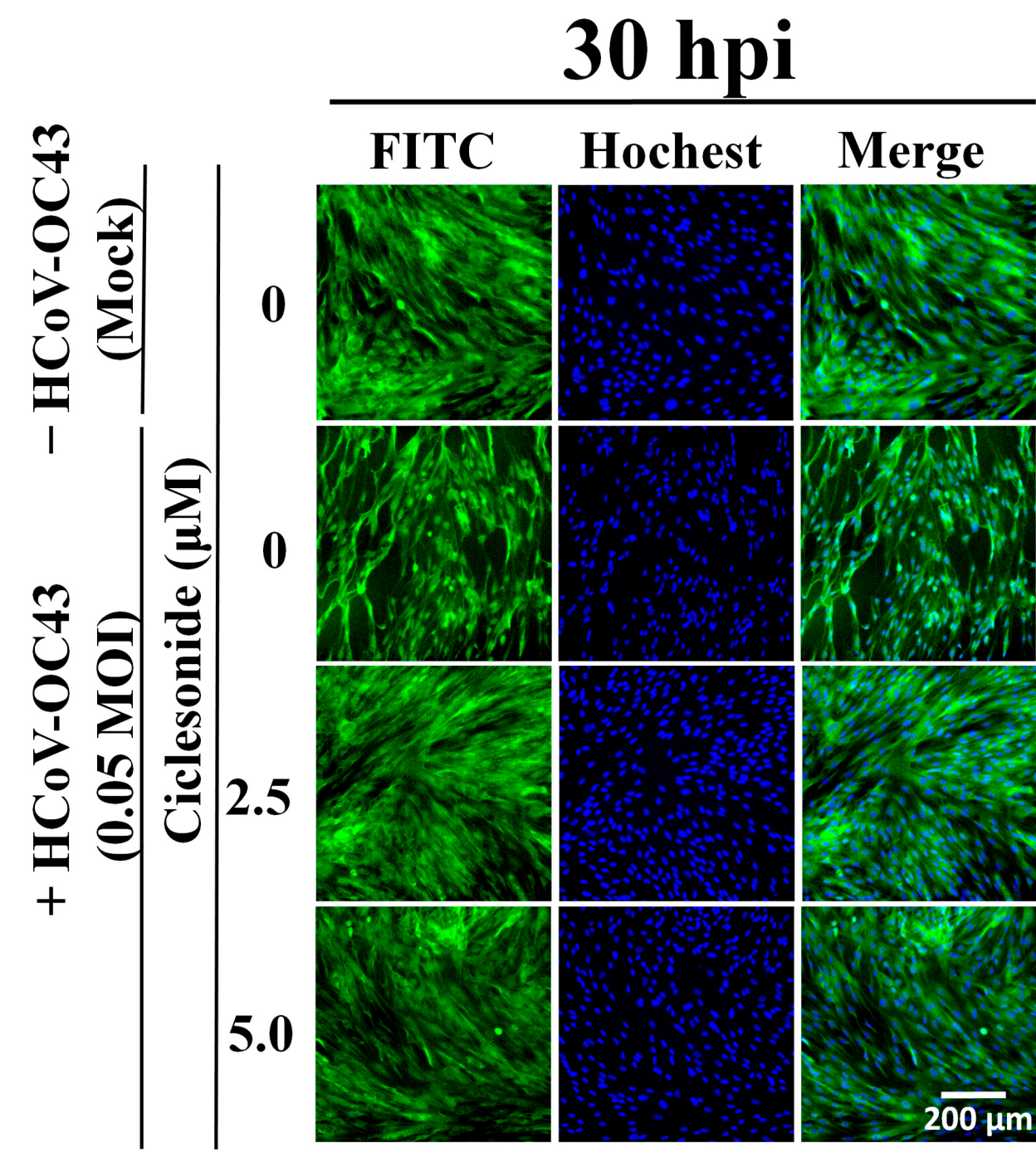

From Figure 3 C.

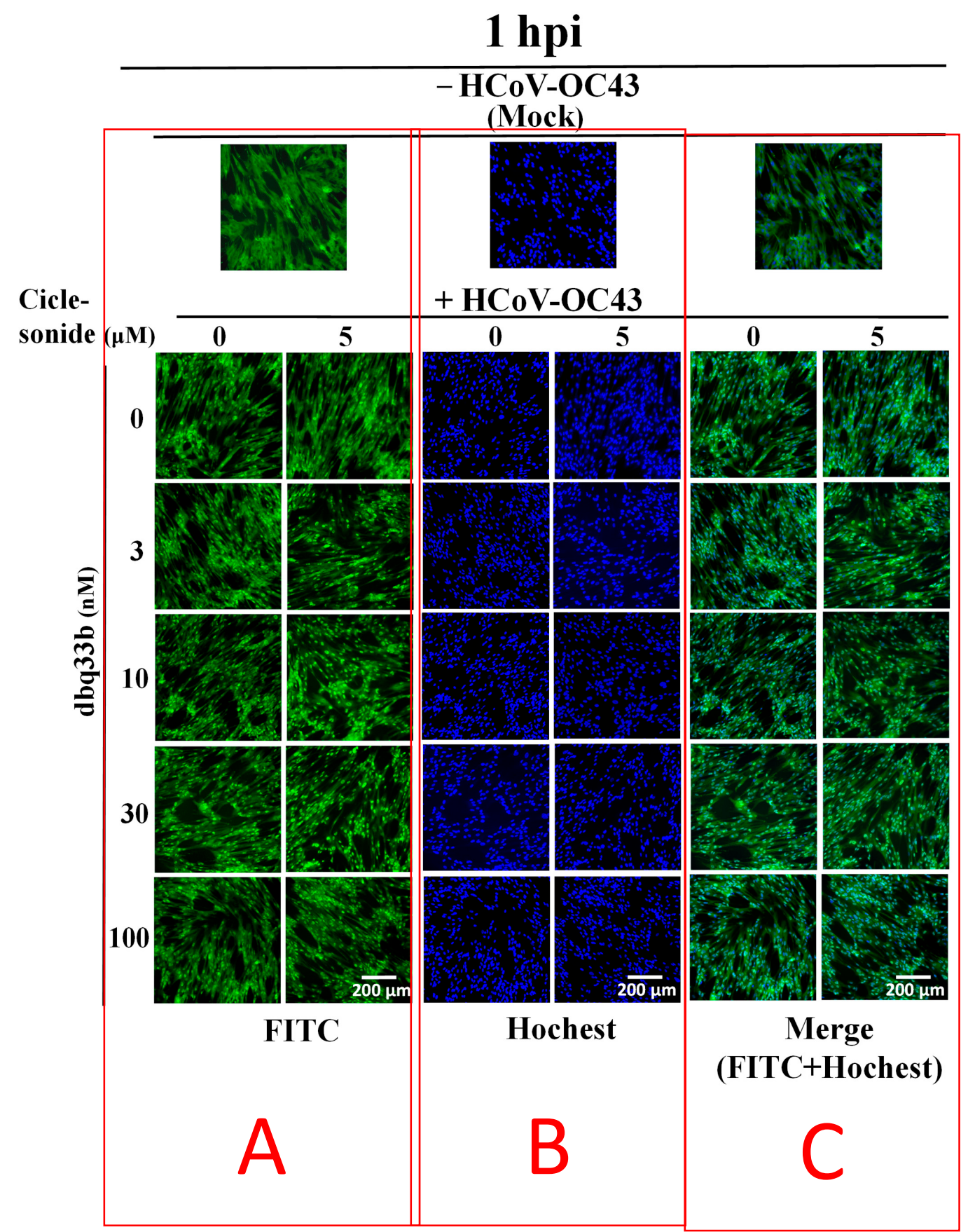

From Figure 3 C.

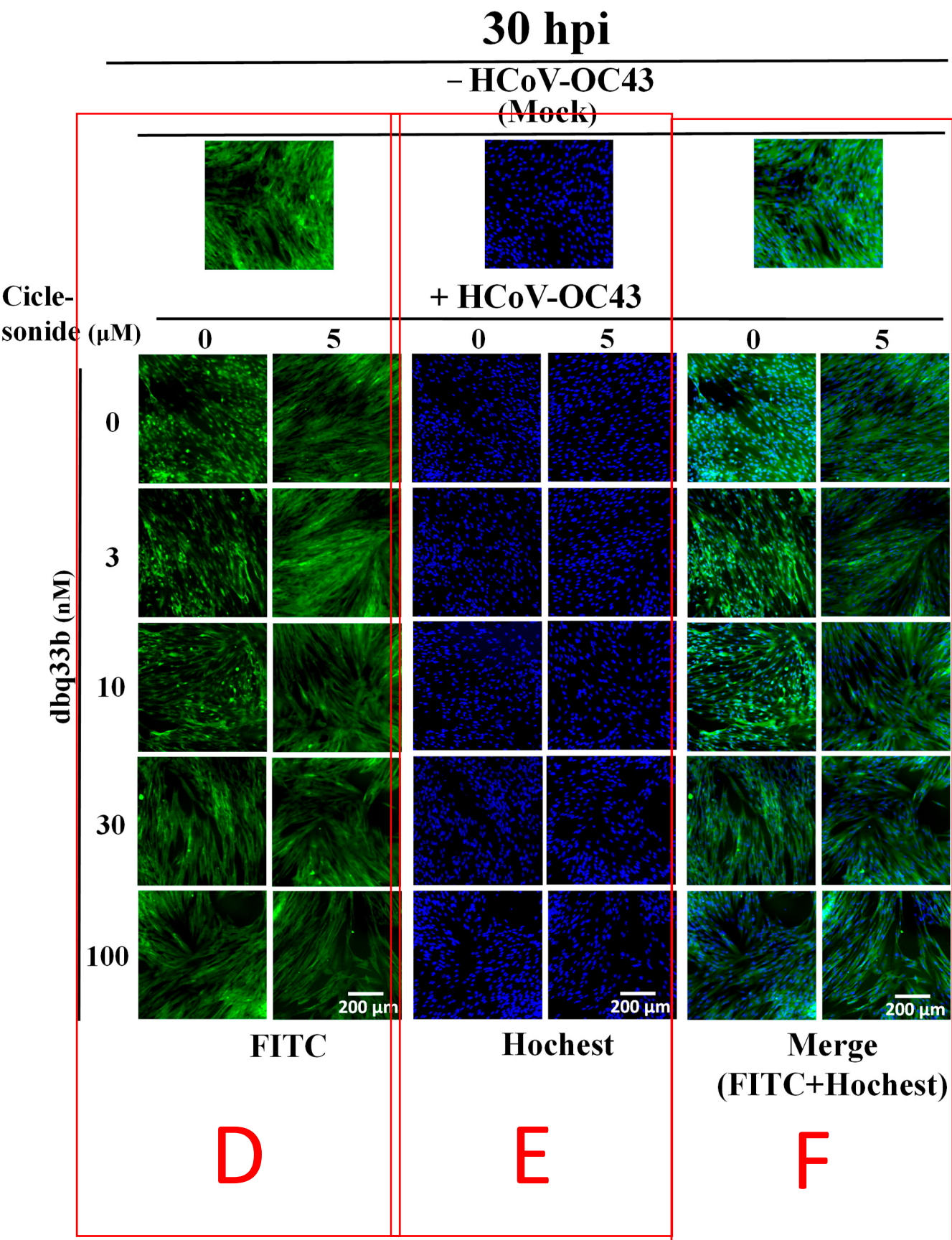

From **A**

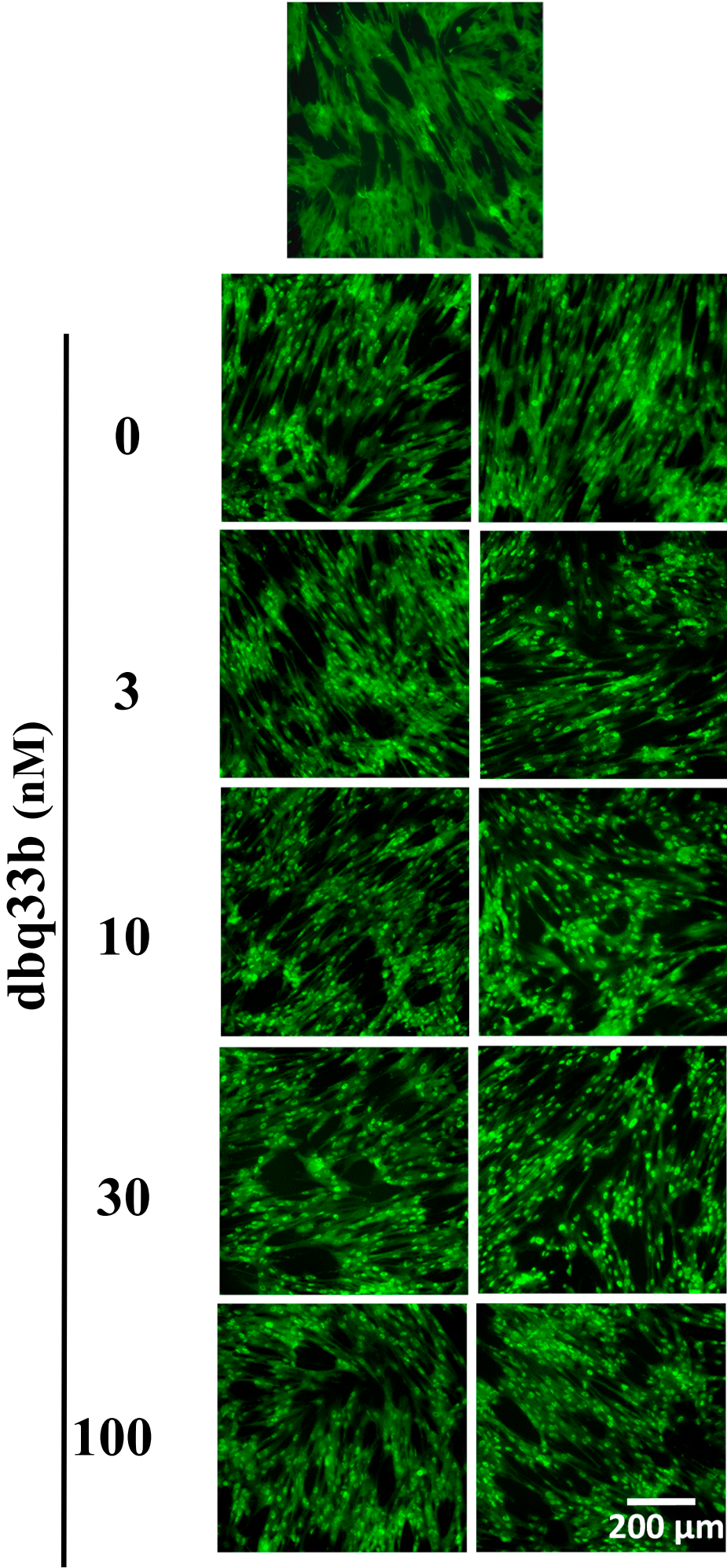

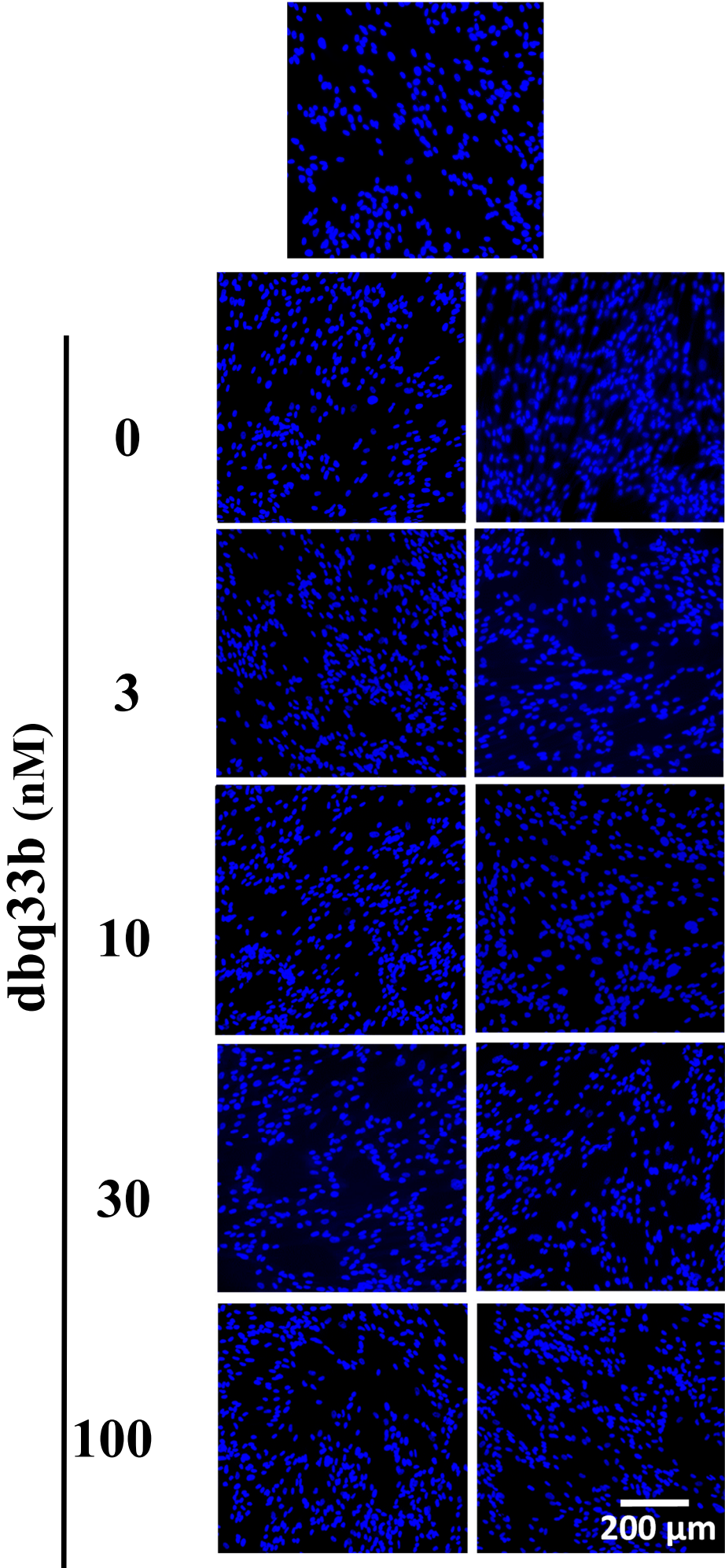

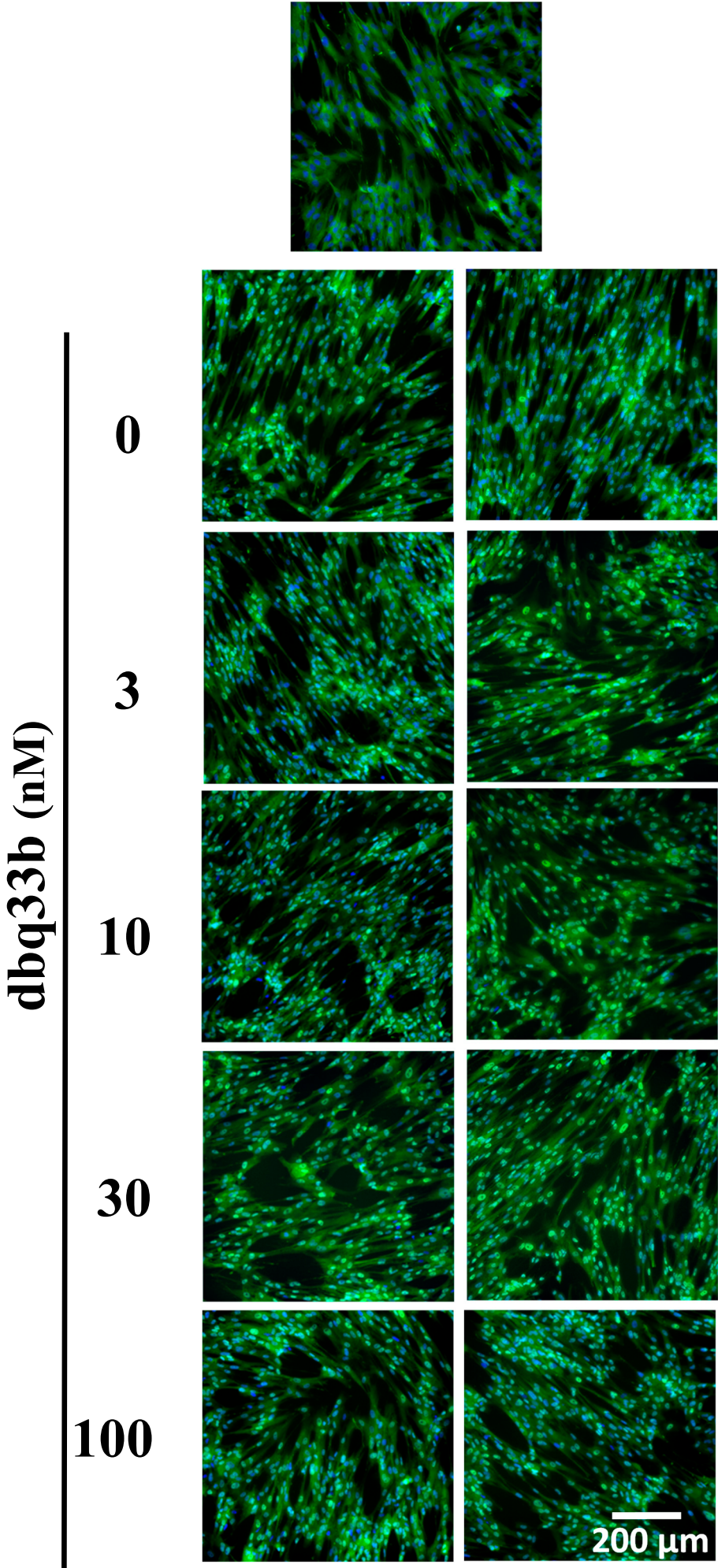

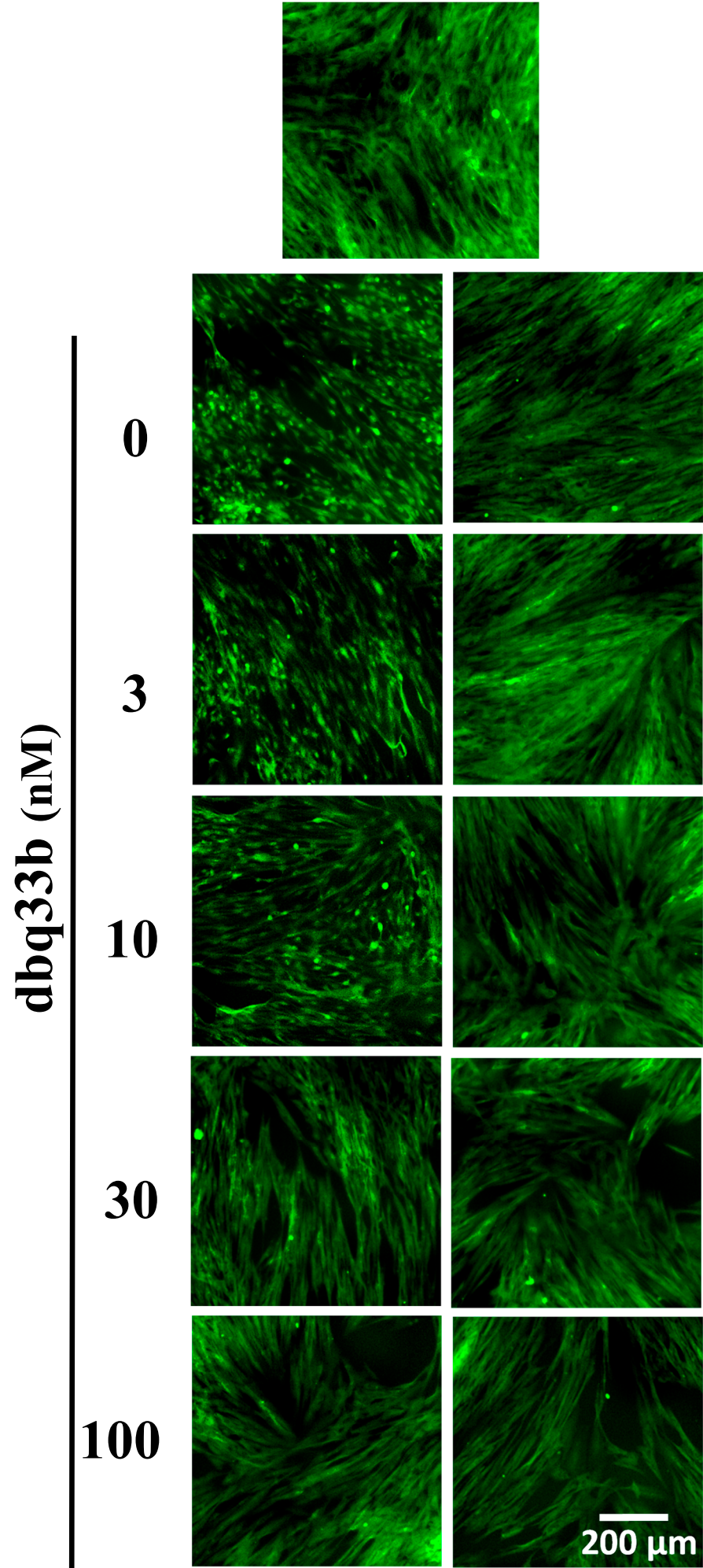

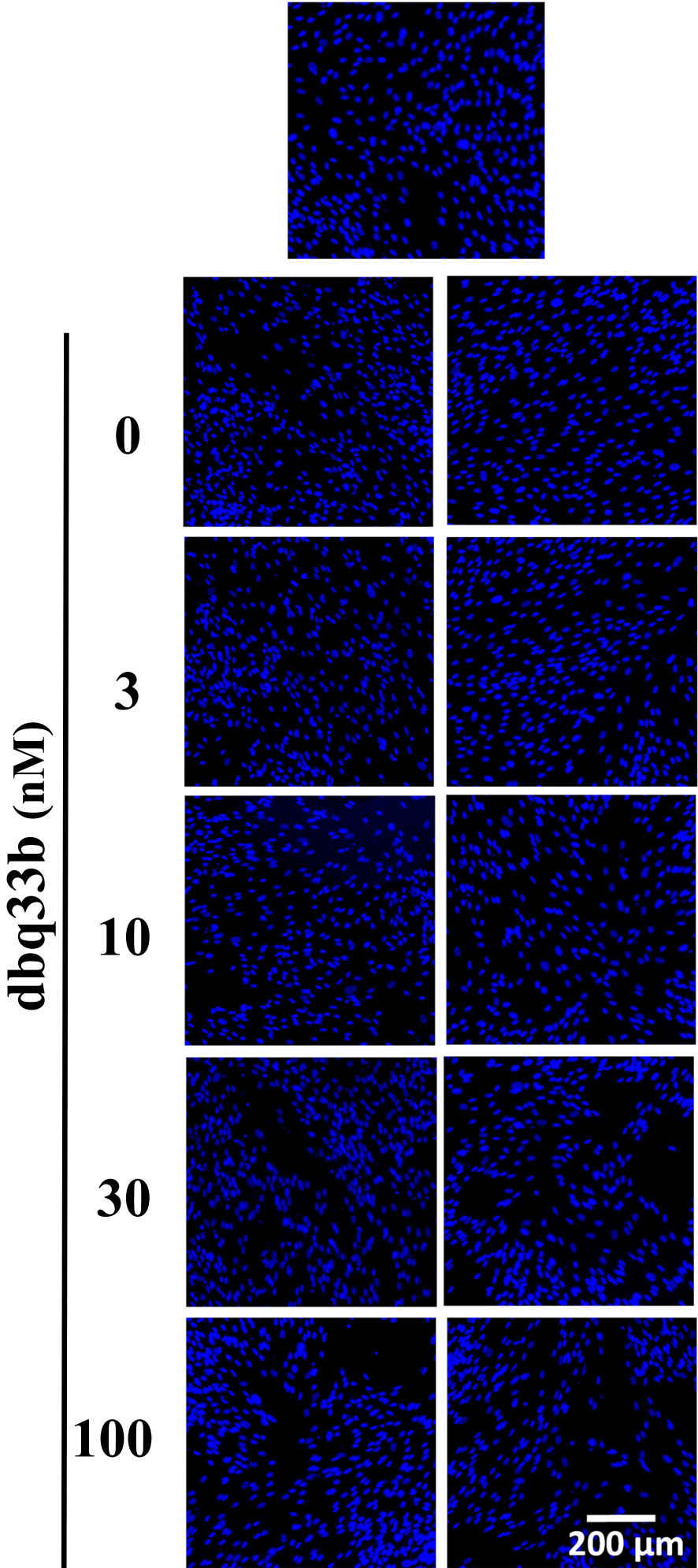

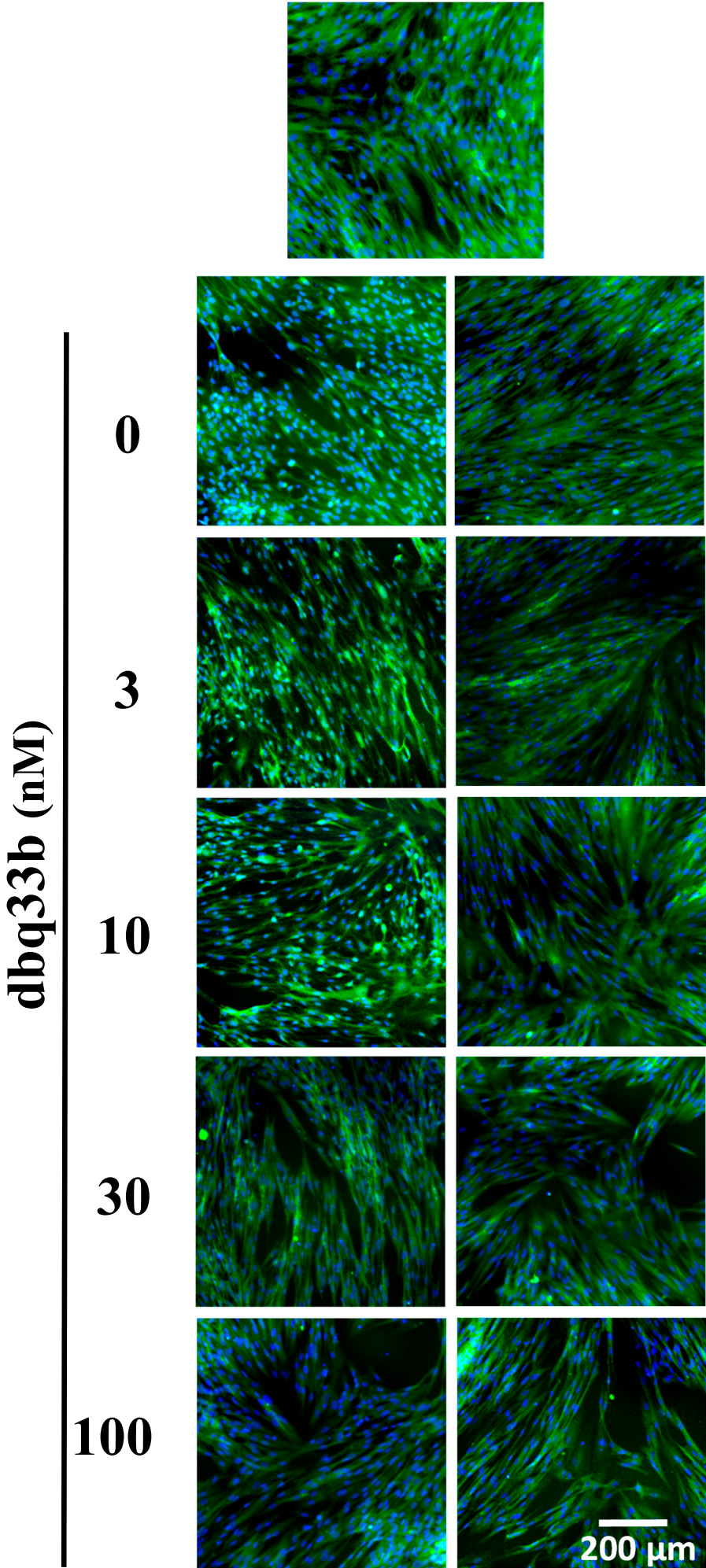

Supplement: Supplementary file 1 [file pharmaceutics-14-01511-s001.zip › pharmaceutics-1775968-supplementary.pdf]
